# Twelve-Year Outcomes of Pterygium Excision with Conjunctival Autograft versus Intraoperative Mitomycin C in Double-Head Pterygium Surgery

**DOI:** 10.1155/2015/891582

**Published:** 2015-02-25

**Authors:** Tommy C. Y. Chan, Raymond L. M. Wong, Emmy Y. M. Li, Hunter K. L. Yuen, Emily F. Y. Yeung, Vishal Jhanji, Ian Y. H. Wong

**Affiliations:** ^1^Hong Kong Eye Hospital, 147K Argyle Street, Kowloon, Hong Kong; ^2^Department of Ophthalmology and Visual Sciences, The Chinese University of Hong Kong, Hong Kong; ^3^Department of Ophthalmology, The University of Hong Kong, Hong Kong

## Abstract

*Purpose*. The study aims to compare the long-term outcome of conjunctival autograft (CAU) and mitomycin C (MMC) in double-head pterygium surgery. *Methods*. This is a follow-up study of a comparative interventional trial. Thirty-nine eyes of the 36 patients with double-head pterygium excision in the original study 12 years ago were recruited for clinical assessment. Seven out of the 36 patients were lost. In the original study, each eye with double-head pterygium was randomized to have pterygium excision with CAU on one “head” (temporal or nasal) and MMC on the other “head.” All patients were invited for clinical assessment for conjunctival bed status and the presence of pterygium recurrence in the current study. *Results*. There was no significant difference between the size, morphology, and type of pterygium among the two treatment groups. The recurrence rate of CAU group and MMC group 12 years after excision was 6.3% and 28.1%, respectively (*P* = 0.020). Among eyes without recurrence, the conjunctival bed was graded higher in the MMC group than the CAU group (*P* = 0.024). *Conclusion*. The use of conjunctival autograft has a significantly lower long-term recurrence rate than mitomycin C in double-head pterygium surgery.

## 1. Introduction

Pterygium is a common degenerative condition of the conjunctiva. It demonstrates an elastotic degeneration of the collagen as a result of excessive ultraviolet exposure [1]. Majority of pterygium occurs in the nasal side, but it is not uncommon to encounter double-head pterygium in the “pterygium belt” region, which locates between 30°N and 30°S of the equator [2]. Pterygium can affect vision by causing tear film instability, inducing corneal astigmatism or blocking the visual axis. Its presence is also a major cosmetic issue. Simple excision of pterygium leaving behind an area of bare sclera has a high recurrence rate ranging from 24 to 89% [3]. Therefore, there are many adopted methods to augment the long-term success of pterygium surgery. Commonly used adjuvants in the literature include conjunctival autograft (CAU) [4], limbal-conjunctival autograft (LCAU) [5], mitomycin C (MMC) [6], and amniotic membrane transplantation (AMT) [7].

LCAU was showed to be superior to MMC in pterygium surgery in a 10-year follow-up study of a randomized controlled trial published recently in the literature [8, 9]. Comparison between CAU and LCAU reported similar success between the two methods in primary pterygium and a superior effectiveness of LCAU in recurrent cases [10]. The present study is a 12-year follow-up study comparing the long-term outcomes and complications of double-head pterygium surgery with CAU to one “head” and MMC application to the opposite “head” of the same eye.

## 2. Methods

This is a follow-up study of a comparative interventional trial, which was approved by the research ethics committees of the Chinese University of Hong Kong and adhered to the tenets of the Declaration of Helsinki. In the original study, 39 eyes of 36 patients with double-head pterygium were randomized to receive CAU to one “head” of the pterygium and MMC application by being defaulted to the opposite “head.” Randomization was done by choosing between two sealed envelopes, labeled “nasal pterygium with CA” in one and “nasal pterygium with MMC” in another. The same surgeon (EY) performed all surgeries at Hong Kong Eye Hospital during the period of May 2000 to June 2001. In this follow-up study, patients in this cohort were invited back to the hospital for clinical examination in September to December 2013 to document the long-term outcomes and complications of the two adjuvants in pterygium surgery. Informed consent had been obtained for every patient before assessment was performed. Clinical examination of the anterior segment and optic disc, intraocular pressure measurement and slit-lamp photography were performed. This follow-up study was approved by the Institutional Review Board of the Hospital Authority.

In the original study, pterygium size and morphology were assessed by the same surgeon (EY). Size of pterygium was measured from limbus to the head of pterygium and the longest diameter was taken. Morphology was assessed using the criteria suggested in the literature [11]. Pterygium was graded into atrophic, intermediate, and fleshy according to the visualization of episcleral vessels underneath the pterygium body with clearly distinguished vessels seen in atrophic type and totally obscured view in fleshy type. The surgeries were performed under retrobulbar anesthesia. Each pterygium head was operated separately with the MMC side operated first. The pterygium and its underlying tissue were excised to achieve a clear margin. Intraoperative MMC (0.02%) was applied directly to the bare sclera using moist vitreous sponges for 5 minutes. The site of MMC application was irrigated thoroughly with at least 50 mL of balanced salt solution. Meticulous care to avoid contamination of MMC to the opposite CAU site was taken. The conjunctiva peripheral to the excised pterygium was then sutured to the episclera. On the pterygium head receiving CAU, a free conjunctival graft was harvested from the superior region of the same eye with dimensions 1 mm larger than the recipient bed. The free graft was then secured to the recipient bed respecting its polarity with interrupted 8.0 polyglactin. Postoperative treatment included a topical steroid (dexamethasone) and antibiotic (chloramphenicol) four times daily for 4 weeks. The first-year result of the original study was presented in a local scientific conference in Hong Kong without publication in the literature.

In the current follow-up study, the main outcome measures included the recurrence rate and residual conjunctival bed status. Recurrence was defined as the presence of fibrovascular proliferation invading the cornea. Conjunctival bed status is graded as A to D [7]. Grade A represents the appearance of the operated site is not different from the normal appearance; grade B represents some fine episcleral vessels in the excised area extend up to but not beyond the limbus and without fibrous tissue; grade C represents additional fibrous tissue is in the excised area but does not invade the cornea; grade D represents fibrovascular tissue invades the cornea and was defined as recurrence in this follow-up study. Two independent assessors (RW and EL), who were blinded to treatment each pterygium received, determined disease recurrence and conjunctival bed grading. A lesion was considered as “recurrence” if one assessor agreed on a disease relapse. As for the conjunctival bed status, the higher grading would be chosen if there was a discrepancy between grading scored by the two assessors. Long-term complications related to CAU or MMC involving the cornea and scleral bed are the secondary outcome measures of the study. Information regarding recurrence and complications by the first postoperative year was traced from the medical records and record of the original study.

Statistical analysis was performed using PASW software version 18.0 (SPSS/IBM, Inc., Chicago, IL). Chi-square and Mann-Whitney *U* test were used to compare qualitative and quantitative variables, respectively, between groups. *P* values of 0.05 or less were considered to be statistically significant.

## 3. Results

There were 39 eyes (78 pterygia) of 36 patients recruited in the original study. The mean follow-up period was 155 ± 4 months (12.9 years). The response rate was 82.1% with 32 eyes (64 pterygia) completing this follow-up study. Six patients (6 eyes) passed away before this follow-up study; one patient (1 eye) was lost to contact. Twenty-seven eyes of 25 patients were assessed in the clinic, while disease recurrence was determined from telephone interview in 4 patients (5 eyes) who were unable to attend the clinic. Supplementary photographs were obtained from those 4 patients for determination of recurrence, but conjunctival bed grading was not performed in them. None of these patients received additional conjunctival surgery after pterygium excision in the study. Demographic and clinical data of patients who completed and defaulted the follow-up study was summarized in Table 1. There was no significant difference in pterygium size (*P* = 0.412), morphology (*P* = 0.251), and type (*P* = 0.792) between the completed and defaulted patients apart from age, which was significantly older in the defaulted patients (*P* = 0.016). Preoperative characteristics of the pterygium between the treatment groups in the current follow-up study were summarized in Table 2. There was no significant difference in size (*P* = 0.403), morphology (*P* = 0.749), and type (*P* = 0.740) of the pterygium between the CAU and MMC groups. Thirteen nasal pterygia were treated with CAU after excision, and 19 nasal pterygia were treated with MMC. The reverse was true for temporal pterygium by default (*P* = 0.134). Moreover, there was no significant difference in size (*P* = 0.512), morphology (*P* = 0.414), and type (*P* = 0.740) between nasal and temporal pterygium.

The Cohen's kappa coefficient, which is a statistical measure of interassessors agreement, was 0.81 signifying almost perfect agreement between the two assessors in the current study. Most recurrences had been observed by the first postoperative year (Table 3). Recurrence of pterygium was noted in 1 case in the CAU group (2.56%) and 6 cases in the MMC group (15.4%) one year after the operation. The difference in recurrence rate was statistically significant between the two treatment groups (*P* = 0.048). Significant difference in recurrence rate was also noted between the two groups 12 years after the pterygium operation. There were 2 cases of recurrence in the CAU group (6.25%), and 9 cases in the MMC group were noted to have disease recurrence (28.1%) (*P* = 0.020). Five recurrent cases were nasal pterygium, and 6 recurrent cases were located on the temporal side (*P* = 740). Among the cases with recurrence observed, all but one were primary pterygium before the operation performed in the study (*P* = 0.434). It was excised and treated with MMC in the original study, but it recurred 3 months afterwards. All the recurrent cases did not undergo further pterygium operations and were managed conservatively according to the patients' preference.

Grading of conjunctival bed was summarized in Table 4. Among the eyes with no disease recurrence on either side (38 pterygia in 19 eyes), conjunctival beds previously treated with MMC were graded higher than the beds covered with CAU in the same eye (*P* = 0.024). Eight eyes showed higher conjunctival bed grades after MMC treatment than that after CAU treatment 12 years after the surgery. The same grades in the two treatment arms were seen in 11 eyes. No eye demonstrated a higher grade after CAU treatment than that after MMC treatment. Difference in conjunctival bed grades was not significant between sites (nasal or temporal) of pterygium (*P* = 0.333).

No severe complication was observed in the first postoperative year. Diffuse punctate epithelial erosions were seen in 8 eyes (20.5%) and all resolved with topical lubricants. Long-term graft survival in the CAU group was excellent. Corneal dellen, conjunctival cyst, pyogenic granuloma, symblepharon, and subconjunctival fibrosis were not observed at sites previously covered with autograft and at the harvest site of CAU. As for the MMC group, areas previously treated with MMC were also free of complications mentioned above. Severe complications including scleral thinning and melting, corneal decompensation, and glaucoma were not detected in any patient attending the follow-up at 12 years after the double-head pterygium surgery.

## 4. Discussion

High recurrence rate is a major problem in pterygium surgery. There are various techniques developed to minimize disease recurrence with CAU and MMC the two most commonly adopted adjuvants. CAU aims at providing immediate coverage of the bare sclera after pterygium excision. This minimizes postoperative inflammation and reduces regrow of the fibrovascular pterygium. Adjuvants including CAU and MMC in pterygium surgery have been summarized in a recently published review article [12]. CAU has been shown to be an effective procedure, with recurrence rates ranging from 2% to 39% after primary pterygium excision [11, 13–15]. On the contrary, MMC is an alkylating agent that prevents cellular activity by inhibiting DNA synthesis. It has antiproliferative effect and prevents recurrence of the pterygium. Previous studies showed recurrence rates varying from 3% to 38% in primary pterygium when MMC was used intraoperatively [6, 16–18]. However, the use of MMC may lead to severe ocular complications including scleral thinning and necrosis, corneal decompensation, and glaucoma [19–22].

The current study had a high response rate with more than 80% of patients participating in the follow-up study. The double-head pterygium in each eye received CAU on one side and MMC on the other as adjuncts, and the pairwise comparison of treatment effects in the same eye minimized interpersonal variability as a confounder. Randomizing the treatment arm to either nasal or temporal pterygium also reduced confounding effect arising from the lesion site. Although there was no difference in the preoperative characteristic of pterygium, such as the size, site, morphology, and type between the 2 study groups, the list of confounding variables is not exhaustive. It is important to note that direct comparison among different studies is difficult because there are variations in surgical techniques including extent of excision and application of MMC, follow-up duration, and definition of recurrence.

The recurrence rate of pterygium after CAU was significantly lower than that of MMC in the current study. Several studies in the literature demonstrated a trend favoring CAU over intraoperative MMC for prevention of pterygium recurrence [15, 16, 23]. Similar findings were observed in a recently published randomized controlled study with 10-year follow-up, which showed a recurrent rate of 6.9% after LCAU and 25.5% after MMC [9]. By including the limbal epithelium in the conjunctival graft, it restores the barrier function of the limbus and helps prevent recurrence. The 10-year recurrence rates after LCAU and MMC reported by Young et al. were similar to the 12-years recurrence rates after CAU and MMC in our study. We may conclude that this is likely the representative recurrence rate for Chinese populations. Reports have shown that both LCAU and CAU were effective in preventing recurrence after pterygium excision, though LCAU showed slight advantage over CAU in recurrence rate in recurrent pterygium [10, 24]. The current study demonstrated a comparable success with CAU in terms of long-term pterygium recurrence rate and the lack of complications such as corneal dellen, conjunctival cyst, pyogenic granuloma, symblepharon, and subconjunctival fibrosis [25].

Conjunctival bed grading was found to be significantly higher after MMC treatment when compared to that after CAU. This finding was consistent with a higher recurrence rate after MMC treatment as shown in current study. Although there was no long-term complication observed between the two adjuvants in this study, adjuvant MMC treatment after pterygium excision was shown to be inferior to CAU in preventing recurrence. It is interested to see that there was an ongoing recurrence in the MMC group (3 eyes) after the first 1 year of pterygium excision, while all the recurrence took place within 1 year in the CAU group. In a 10-year follow-up study by Young et al., there was 1 recurrent case in the LCAU group and 3 recurrent cases in the MMC group after the first postoperative year [9]. On the contrary, Koranyi et al. did not observe any recurrence after the 12 months visit in a 4-year comparative study between CAU and MMC in primary pterygium surgery [23]. Survival curve analysis also showed that there was a 97% chance that there would be a recurrence within 1 year of pterygium removal [26]. Such difference in our observation remained to be elucidated. The ongoing recurrence observed could be the result of persistent ocular inflammation or irritation in the site previously managed with intraoperative MMC while leaving the bare sclera behind [1]. Similar to the study design of Young et al. [9], we were not able to identify the exact time of pterygium recurrence during the extended follow-up period because all the patients were discharged from the original study (1 year) before they were invited back for the current follow-up study. Nevertheless, majority of the recurrence cases occurred within the first postoperative year; this may signify the need to monitor patient who underwent pterygium excision for at least a year before discharge.

Moreover, this study was also a noncomparative analysis of using CAU and MMC in double-head pterygium surgery. In the current study, recurrence was found in 28% of our cases (9 of the 32 eyes). Two eyes had recurrence over both heads, and 7 eyes had recurrence over either head treated with MMC previously. Other options in double-head pterygium surgery included rotational CAU [27], split-CAU [28], sequential CAU [29], MMC [30], and AMT [31]. All of these treatments aimed at preventing recurrence despite the larger conjunctival defects remained after removal of the two heads. A previous interventional cohort in our hospital involved combining rotational CAU and CAU. It demonstrated a recurrence rate of 35% in 20 eyes [27]. In that study, rotational CAU was harvested from the larger pterygium and placed over the conjunctival defect of the smaller pterygium with 180-degree rotation. The defect of the larger pterygium was then covered with CAU harvested from the superior bulbar conjunctiva.

Another way to cover the bare sclera was using split-CAU. Split-CAU aimed at covering both conjunctival defects with a large CAU divided from the superior bulbar conjunctiva. No recurrence was found in a retrospective evaluation of 7 eyes over a mean follow-up period of 18 months [28]. However, adequate exposure may be difficult in small Chinese eyes. By performing sequential CAU to each head separately, a Canadian group showed only 1 nasal recurrence (5.6%) after 2 years in a retrospective study of 9 eyes [29]. This allowed CAU to each head but avoiding extensive CAU dissection. On the other hand, MMC and AMT are alternatives in eyes when CAU is not feasible. Intraoperative MMC application was used solely after double-head pterygium excision in a case series of 13 eyes. In this case series, 1 eye (8.0%) had recurrence in a follow-up period of 3 years [30]. Similar recurrence rate (9.1%) was shown with AMT after extensive conjunctival excision of 11 eyes observed for 1 year [31]. Although the results of our study appeared to be inferior to other studies in the literature, most studies were limited by the small sample size, short follow-up duration, and retrospective nature, making direct comparison difficult among them. This is understandable as the reported incidence of double-head pterygium was less than 3%, making case recruitment difficult [32]. The current method we adopted for double-head pterygium surgery is combining CAU with rotational CAU or AMT to cover the bare sclera.

In conclusion, both CAU and MMC were shown to be safe adjuvants in pterygium surgery. CAU appeared to be a better choice with lower recurrence rate and better conjunctival appearance when compared to MMC. Bare sclera pterygium excision in the presence of adjuvant MMC should not be performed given the significantly high rate of long-term recurrence.

## Figures and Tables

**Table 1:** Demographic and Clinical Data of Patients with double head pterygium.

	All (*n* = 39)	Completed (*n* = 32)	Defaulted (*n* = 7)	*P* values
Age (years)	60.9 ± 10.1	59.2 ± 10.2	68.9 ± 5.21	0.016^a^
Gender (M : F)	24 : 15	19 : 13	5 : 2	0.553^b^

	All (*n* = 78)	Completed (*n* = 64)	Defaulted (*n* = 14)	*P* values

Mean size of pterygium (mm)	2.63 ± 0.69	2.59 ± 0.71	2.71 ± 0.61	0.412^a^
Morphology of pterygium				0.251^b^
Atrophic	19 (24.4%)	15 (23.4%)	4 (28.6%)	
Intermediate	40 (51.3%)	31 (48.4%)	9 (64.3%)	
Fleshy	19 (24.4%)	18 (28.1%)	1 (7.14%)	
Pterygium type				0.792^b^
Primary	65 (83.3%)	53 (82.8%)	12 (85.7%)	
Recurrent	13 (16.7%)	11 (17.2%)	2 (14.3%)	

CAU = conjunctival autograft; MMC = mitomycin C.

^a^Mann-Whitney *U* test between completed and defaulted groups.

^b^Chi-square test between completed and defaulted groups.

**Table 2:** Preoperative Characteristics of Pterygium in the Conjunctival Autograft and Mitomycin C Groups Who Completed 12-tear-follow-up.

	CAU (*n* = 32)	MMC (*n* = 32)	*P* values
Size of pterygium (mm)	2.53 ± 0.72	2.66 ± 0.70	0.403^a^
Site of pterygium			0.134^b^
Nasal	13 (40.6%)	19 (59.4%)	
Temporal	19 (59.4%)	13 (40.6%)	
Morphology of pterygium			0.749^b^
Atrophic	7 (21.9%)	8 (25.0%)	
Intermediate	17 (53.1%)	14 (43.8%)	
Fleshy	8 (25.0%)	10 (31.3%)	
Pterygium type			0.740^b^
Primary	26 (81.3%)	27 (84.4%)	
Recurrent	6 (18.8%)	5 (15.6%)	

CAU = conjunctival autograft; MMC = mitomycin C.

^a^Mann-Whitney *U* test between CAU and MMC groups.

^b^Chi-square test between CAU and MMC groups.

**Table 3:** Total Number of Recurrences in Conjunctival Autograft and Mitomycin C Groups.

	CAU	MMC	*P* values^a^
Recurrence by 3 months (*n* = 39 in each group)	0	1 (2.56%)	0.314
Cumulative recurrence by 6 months (*n* = 39 in each group)	1 (2.56%)	5 (12.8%)	0.089
Cumulative recurrence by 1 year (*n* = 39) in each group	1 (2.56%)	6 (15.4%)	0.048
Cumulative recurrence by 12 years (*n* = 32) in each group	2 (6.25%)	9 (28.1%)	0.020

CAU = conjunctival autograft; MMC = mitomycin C.

^a^Chi-square test between CAU and MMC groups.

**Table 4:** Comparison of Conjunctival Bed Grading in Conjunctival Autograft and Mitomycin C Groups.

	CAU	MMC	*P* values^a^
Conjunctival bed grade (*n* = 19) in each group			0.024
Grade A	14 (73.7%)	6 (31.6%)	
Grade B	5 (26.3%)	11 (57.9%)	
Grade C	0	2 (10.5%)	
Recurrence (*n* = 32) in each group			
Grade D	2 (6.25%)	9 (28.1%)	0.020

CAU = conjunctival autograft; MMC = mitomycin C.

^a^Chi-square test between CAU and MMC groups.
